# Process audit of pain assessment recording in hospitalised canine ophthalmology patients

**DOI:** 10.18849/ve.v10i2.705

**Published:** 2025-05-02

**Authors:** Vicky Lilley, Niamh Clancy

**Keywords:** analgesia, canine, clinical audit, communication, improving practice, ocular, opthamology, pain assessment, record keeping

## Abstract

**Aims and objectives:** The aim of this process audit was to assess if pain assessment in hospitalised canine ophthalmology patients was being documented and if found to be undocumented, implementing changes to improve this.

**Background:** Pain scoring anecdotally appeared to be recorded less in ophthalmology patients than in other patients in the practice. This was of particular concern to veterinary staff working out of hours shifts who rely on hospital records to monitor changes in patient condition. The target was for all patients to have a pain assessment recorded.

**Methods:** The records of 30 patients were analysed to find out how many had a pain score chart completed when requested or if they had any notes regarding pain assessment recorded on their hospital sheet.

**Results:** The initial audit data collected confirmed the suspicion that pain scoring was not adequately recorded in hospitalised canine ophthalmology patients, with 57% of patients having no pain assessment recorded.

**Implementation of changes (team discussion & changes made) and re-audit:** One month after implementing interventions that required staff to record signs of ocular pain on the patients hospital sheet, and the development of a mnemonic to remind staff of these signs and as the placement of reminder signs in prominent areas, a re-audit was performed. This showed significant improvement; 96% of patients had a pain assessment recorded.

**Application:** Performing this clinical audit enabled the practice to identify an area where improvements needed to be made. The team collaborated to devise a set of interventions they felt would address this issue improved the recording of pain assessment in this practice. This has allowed staff on differing shifts to recognise changes promptly and ultimately improve the wellbeing of patients. Other practices may find the interventions useful and wish to perform a similar audit.

## Introduction

Since their regulation in 2012, veterinary nurses have been required to adhere to clinical governance standards (RCVS, 2012; RCVS, 2015; RCVS, 2020). Recently, they have also been tasked with reflecting on their learning activities when documenting continuing professional development (RCVS, 2023. One way to fulfill both of these requirements is by conducting a clinical audit and reflecting on the process. Furthermore, such audits can positively impact clinical standards, team performance, and patient care. This audit aimed to assess and improve the extent to which hospitalised canine ophthalmology patients' pain assessments were recorded. The target was for all patients to have a pain assessment recorded during their hospitalisation. It is well recognised that recognition of pain is an essential skill in veterinary medicine and that pain scoring is likely to assist in the recognition of pain in pet animals (Crompton, 2010; Hunt, 2014; Thornley, 2015; Bloor & Allan, 2017; Tomlinson & Reynolds, 2018; Hernandez-Avalos et al., 2019; Gruen et al., 2022).

Although the short form of the Glasgow Composite Pain Score (SF-GCPS) is routinely used for pain scoring in the author's practice, it was observed anecdotally that this assessment was less frequently completed for hospitalised ophthalmology patients. The lack of documented pain assessments is particularly concerning for staff working shifts, as they rely on written hospital records to monitor changes in patient condition when it may not be possible to communicate with the previous shift's staff. This process clinical audit aimed to confirm or refute these anecdotal observations. Subsequently, the audit confirmed a deficiency in pain assessment recording for this patient group, leading to a meeting to address the underlying reasons. Following this, changes were implemented to enhance communication and, ultimately, improve patient care.

## Methods

All patients receiving pain relief should have their pain levels assessed to monitor treatment effectiveness. This has basis in literature which states pain assessment should be repeated regularly to assess response to treatment given (Crompton, 2010; Thornley, 2015; Bloor & Allan, 2017). However, it could be suggested that all ophthalmic patients should have a pain assessment due to there being a risk for pain. The International Society of Feline Medicine state that a pain assessment should be performed in cats at every physical examination to prevent pain being overlooked (Steagall et al,. 2022); therefore, it can be assumed that this should also be the case for canine patients. This assumption is supported by Thurston (2020) who stated that all patients should be assessed for pain regardless of what condition they have. With this in mind, the target for improvement was for all hospitalised canine ophthalmology patients to have a pain assessment recorded during their stay.

The author, who is a registered veterinary nurse (RVN), alongside the head surgery ward RVN, and the ophthalmology RVN, formed the team responsible for driving change during this audit. Data was collected retrospectively to prevent accidental changes in staff behaviour during the initial audit cycle due to awareness of the audit taking place. Initial data was collected to show the number of ophthalmic patients who had a SF-GCPS requested on their hospital sheet for completion, the number of requested SF-GCPS that were recorded and if any other notes regarding pain were recorded during the patients stay. A team meeting was held to disseminate the initial findings and develop a set of interventions. After implementing these interventions , a re-audit was performed to measure any improvement.

To identify all admitted canine ophthalmology patients, the hospitals digital diary was used. Each service has its own specific area within this booking system. The author was able to locate all admitted patients by going through the diary day by day. In this practice, when the patient leaves the hospital its kennel charts and all accompanying paperwork (such as SF-GCPS charts) are scanned and uploaded onto its computerised record by the administration team. Day patients (who are not managed exclusively by the ward nurses and may be having non-painful diagnostics) and those with incomplete records (some were missing pages; therefore, it could not be determined if a pain assessment was on the missing pages) were excluded. The remaining records are then able to be analysed. A spreadsheet was used to record if the patient had a SF-GCPS requested, if that pain score was recorded and if any notes were made regarding pain during the patient’s hospital stay.

Each patient record was looked at as a single data set rather than looking at each day the patient was in or each time a pain score was requested. Therefore, a single completed pain score or note on the kennel sheet recording ophthalmic pain descriptors at any time during the patients stay was marked on the spreadsheet as a positive result and no further data was collected from that patient.

This practice has an ophthalmology referral service, so it has a high case load of both elective and emergency surgical patients, and emergency medical management patients. The author wanted to ensure all three types were captured during the audit period so opted to collect data for the full two-month period to ensure this was the case. This was collected during the months of April and May 2022. For the re-audit the data was collected until a similar range and number of patient data was achieved this ended up being a six week period from the 20 July 2022 onward.

## Results

The initial audit included data from 30 patient records over 2 months. Of these:

- 43% (13/30) had either a recorded pain score or pain assessment notes written on their charts.
- 43% (13/30) had a pain score requested on their charts.
- 38% (5/13) of those marked for a pain score had one recorded.
- 62% (8/13) of those marked for a pain score had pain assessment notes written on their charts.
- 0% (0/17) of the remaining patients had pain assessment notes recorded on their charts.

These results confirmed recording of pain assessment in the group was unacceptably low. Interventions would be needed to reach the target of all patients receiving a pain assessment recorded during their hospitalisation period.

A meeting was held to discuss the results and formulate a plan to improve these results. The author led the meeting and formulated an intervention plan with input from the team.

A separate audit to address missing documentation would be advised. This is not performed by the author's department and therefore was not included in the recommendations recorded here.

The author was responsible for implementing the action plan, with support from the head nurse.

The outcome of the initial audit and meeting were circulated to all ward nurses to comment on and add additional feedback if they had any.

Nurses were reminded by the head nurse in team meetings that performing a pain score when requested is practice policy and should be adhered to. Reminder notices were displayed in prominent locations. This was done immediately following the meeting.

Reminders of ophthalmic pain descriptors were to be distributed for use when recording written pain assessments on hospital charts. The author performed a literature search to find these and created an acronym (supplementary material 1) that was displayed in prominent areas to help staff remember the need to record pain scores.

## Implementation of changes (team discussion & changes made)

Nursing interventions/action plan developed as a team: Nurses were reminded they must complete a pain score if one is requested, as this is practice policy. Nurses will record signs of ocular pain as documented in the literature (Stiles et al., 2003; Park et al., 2010; Clark et al., 2011; Thomson et al., 2013; Knott, 2020; Foote, 2021; Lee & Yon, 2021; Ortolani et al., 2021) on the patient’s hospital sheet at least once per shift or whenever opioids are given to monitor treatment effectiveness. This will be in addition to the completion of a pain score chart if one has been requested. Ocular pain signs may present differently to other forms of pain; therefore, an acronym to remember signs of ocular pain discussed in the literature (Stiles et al., 2003; Park et al., 2010; Clark et al., 2011; Thomson et al., 2013; Knott, 2020; Foote, 2021; Lee & Yon, 2021; Ortolani et al., 2021) was developed (supplementary material 1) and signs displaying this were placed around the ward as a reminder. The idea that it would spell out the beginning of the word blephorospasm (BLEPH) was used as blephorospasm was the most commonly cited indicator of ocular pain. Reminder notes regarding completion of pain scores were affixed to the dangerous drugs cupboard as this is where opioid drugs are collected from. Both notes were also displayed on the ward’s reminders noticeboard. A plan was made to perform a re-audit after the interventions had been in place for one month to assess how these interventions were working.

Intervention outcomes: By allowing the nurses to make notes specific to ocular pain, more regular assessments and recordings were carried out, regardless of whether a pain score had been specifically requested. This allowed nurses on different shifts to compare their assessments with what had been noted previously and alert the veterinary surgeon to any changes. Fewer patients had pain score requests on their hospital sheet in the re-audit than in the initial audit: 37% (10/27) compared to 48% (13/30). However, the requesting of pain scores is not what this audit was focused on, rather the completion of requested scores and notes. Pain score chart completion rates increased to a 90% (9/10) completion rate compared with 38% (5/11) pre-intervention. Overall recording of pain assessment (either a completed chart or pain assessment notes written) improved from a 43% (13/30) recording rate to a 96% (26/27) recording rate post interventions.

Barriers encountered: A meeting was held virtually on Microsoft Teams (video conferencing software) to discuss the audit results. Out of nineteen staff members, the author and sixRVNs attended, while twelve RVNs were unable to attend due to shift patterns and needing to cover the ward. The main barrier to completing a written pain assessment appeared to be the pain scoring method used. It was felt this pain scoring system was not relevant to ocular pain signs and therefore was not completed. Nurses stated they would directly inform the veterinary surgeon about suspected pain rather than complete paperwork. It was discussed why having a paper record of pain assessment was necessary to help inform those on the following shifts of any change in pain levels. Further ideas to help reach the target were encouraged and developed during this meeting. However, despite using a virtual platform that in theory meant as many people as possible could attend, low attendance meant not everyone was involved in this discussion. Meeting notes were circulated afterwards and any additional ideas would have been considered, although no more were presented. In future, combining the mid-audit meeting with one of the compulsory in-person team meetings may help attendance.

Introduction of an ocular pain score would have been ideal as the SF-GCPS is not validated for ocular pain and evidence suggests that ocular pain scales are more effective in recognising ocular pain (Stiles et al., 2003; Park et al., 2010; Clark et al., 2011; Thomson et al., 2013; Knott, 2020; Foote, 2021; Lee & Yon, 2021; Ortolani et al., 2021). However, it was not possible to make this change at this time due to the hospital awaiting the results of research into this area. Another option discussed was that of fully computerised records that enforce pain score completion prior to being able to mark treatment as complete. This cannot be introduced immediately but may be possible in the future.

## Re-audit results

The methodology used for the re-audit was unchanged from the initial audit.

27 patients’ data was analysed over a six week period, one month after the implementation of interventions, and results showed significant improvements:

- 96% (26/27) of patients had either a completed pain score chart or pain assessment notes recorded during their stay, compared to 43% (13/30) in the initial audit.
- 37% (10/27) had a pain score requested on their charts.
- Of those that had a pain score requested 90% (9/10) had this completed, compared to 38% (5/13) in the initial audit.
- 90% (9/10) of those marked for a pain score also had additional pain assessment notes recorded compared to 62% (8/13) in the initial audit.
- 100% (17/17) of the remaining patients had pain assessment notes recorded on their kennel charts.

## Conclusion

This was the first clinical audit the author had performed so there was a learning curve for performing the audit itself as well as for leading change. As a direct result of performing this audit, the author feels they have become a more effective leader and would not hesitate to perform another audit in the future. For this audit, it is likely a repeat audit would be useful to see if the interventions are being adhered to as time passes. Once the new hospital record system is implemented or new evidence on ocular pain scores arises, further interventions can be made and then audited again.

## Application

This audit has improved the recording of pain assessment in this practice. This improved communication has allowed those on differing shifts to recognise changes promptly and ultimately improve the wellbeing of their patients. A second re-audit cycle is recommended in the future to see if standards are being upheld as time passes. Changes to kennel charts are in planning stages, and once changed a re-audit should take place to assess how these changes affect the recording of pain assessment. Other practices may find the interventions useful and wish to perform a similar audit.

## Ethical approval

Ethical approval was not required because this was a clinical audit carried out in an individual practice for the purpose of quality improvement (RCVS, 2023), a process recommended in the RCVS Code of Professional Conduct for Veterinary Nurses (RCVS, 2020). No identifying data was recorded for any patient or client.

## Informed consent

Informed consent was obtained from the hospital director to publish this audit. However, no-one is identifiable.

## Supplementary materials

Supplementary material S1 – Supplementary 1: Acronym reminder sheet.

### Author contributions

**Vicky Lilley**: Conceptualisation, Methodology, Investigation, Formal Analysis, Project Administration, Writing - Original Draft, Writing - Review and Editing. **Niamh Clancy**: Writing - Review and Editing, Supervision.

### Acknowledgements

This clinical audit was completed as part of my studies for my Post Graduate Certificate in Advanced Veterinary Nursing (Anaesthesia and Analgesia pathway) run by the School for Veterinary Nursing at the Royal Veterinary College. I would like to thank all of the CertAVN teaching staff for teaching me how to review literature and perform this audit! I would never have done it without them. I would also like to thank all of my colleagues for giving me their time and ideas and helping improve things for our patients.

### ORCiD

Vicky Lilley: https://orcid.org/0000-0002-9587-9104

Niamh Clancy: https://orcid.org/0000-0002-3674-4736

### Conflict of Interest

The authors declare no conflicts of interest.

## References

[BIBR-1] Bloor C., Allan L. (2017). Pain scoring systems in the canine and feline patient. The Veterinary Nurse.

[BIBR-2] Clark J.S., Bentley E., Smith L.J. (2011). Evaluation of topical nalbuphine or oral tramadol as analgesics for corneal pain in dogs: a pilot study. Veterinary Ophthalmology.

[BIBR-3] Crompton S. (2010). Pain assessment and pain scoring models: a review. The Veterinary Nurse. 10.12968/vetn.2010.1.1.22.

[BIBR-4] Foote A. (2021). Evaluation of acute and chronic ophthalmic pain. Veterinary Nursing Journal.

[BIBR-5] Gruen M.E., Lascelles B.D.X., Colleran E., Gottlieb A., Johnson J., Lotsikas P., Marcellin-Little D., Wright B. (2022). 2022 AAHA Pain Management Guidelines for Dogs and Cats. Journal of the American Animal Hospital Association.

[BIBR-6] Hernandez-Avalos I., Mota-Rojas D., Mora-Medina P., Martínez-Burnes J., Casas Alvarado A., Verduzco-Mendoza A., Lezama-García K., Olmos-Hernandez A. (2019). Review of different methods used for clinical recognition and assessment of pain in dogs and cats. International Journal of Veterinary Science and Medicine.

[BIBR-7] Hunt J. (2014). Pain assessment in small animal practice. Companion Animal.

[BIBR-8] Knott T. (2020). Ophthalmology for RVNs. The Veterinary Nurse. 10.12968/vetn.2020.11.2.56.

[BIBR-9] Lee L., Yon E. (2021). Pain management and intraocular pressure monitoring following phacoemulsification. The Veterinary Nurse.

[BIBR-10] Ortolani F., Scilimati N., Gialletti R., Menchetti L., Nannarone S. (2021). Development and preliminary validation of a pain scale for ophthalmic pain in horses: The Equine Ophthalmic Pain Scale (EOPS. The Veterinary Journal.

[BIBR-11] Park S.A., Park Y.W., Son W.G., Kim T.H., Ahn J.S., Ahn J.T., Kim S.E., Lee I., Seo K. (2010). Evaluation of the analgesic effect of intracameral lidocaine hydrochloride injection on intraoperative and postoperative pain in healthy dogs undergoing phacoemulsification. American Journal of Veterinary Research.

[BIBR-12] (2012). Cracking the codes. RCVS News.

[BIBR-13] (2015). Know your Code: Veterinary nursing and clinical governance. Veterinary Nursing Journal.

[BIBR-14] (2020). 6. Clinical governance [Code of Professional Conduct for Veterinary Nurses: Supporting guidance, Section 6]. RCVS. https://www.rcvs.org.uk/setting-standards/advice-and-guidance/code-of-professional-conduct-for-veterinary-nurses/supporting-guidance/clinical-governance/.

[BIBR-15] (2023). CPD Policy and Guidance for VNs. https://www.rcvs.org.uk/document-library/cpd-policy-for-vns/.

[BIBR-16] Knowledge R.C.V.S. (2023). Clinical Audit – addressing ethical concerns. https://knowledge.rcvs.org.uk/document-library/clinical-audit--addressing-ethical-concerns/.

[BIBR-17] Steagall P.V., Robertson S., Simon B., Warne L.N., Shilo-Benjamini Y., Taylor S. (2022). 2022 ISFM Consensus Guidelines on the Management of Acute Pain in Cats. Journal of Feline Medicine and Surgery.

[BIBR-18] Stiles J., Honda C.N., Krohne S.G., Kazacos E.A. (2003). Effect of topical administration of 1% morphine sulfate solution on signs of pain and corneal wound healing in dogs. American Journal of Veterinary Research.

[BIBR-19] Thomson S.M., Oliver J.A., Gould D.J., Mendl M., Leece E.A. (2013). Preliminary investigations into the analgesic effects of topical ocular 1% morphine solution in dogs and cats. Veterinary Anaesthesia and Analgesia.

[BIBR-20] Thornley A. (2015). Consideration of pain scoring systems. The Veterinary Nurse. 10.12968/vetn.2015.6.10.613.

[BIBR-21] Thurston A. (2020). How to implement and use post-operative pain scoring systems effectively in general practice. Veterinary Nursing Journal.

[BIBR-22] Tomlinson E., Reynolds H. (2018). A comparative study analysing two pain score scales pre and post operatively on felines undergoing surgery for ovariohysterectomy. The Veterinary Nurse.

